# Determination of the Time-frequency Features for Impulse Components in EEG Signals

**DOI:** 10.1007/s12021-024-09698-y

**Published:** 2025-01-23

**Authors:** Natalia Filimonova, Maria Specovius-Neugebauer, Elfriede Friedmann

**Affiliations:** 1 Biology and Medicine Institute Science Educational Center, Taras Shevchenko National University of Kyiv, Volodymyrska St, 60, Kyiv, 01033 Ukraine; 2 Institute of Mathematics, University of Kassel, Heinrich-Plett-Str. 40, Kassel, 34132 Germany

**Keywords:** Visual system, Wavelet analysis, Krawtchouk functions, EEG processing

## Abstract

Accurately identifying the timing and frequency characteristics of impulse components in EEG signals is essential but limited by the Heisenberg uncertainty principle. Inspired by the visual system’s ability to identify objects and their locations, we propose a new method that integrates a visual system model with wavelet analysis to calculate both time and frequency features of local impulses in EEG signals. We develop a mathematical model based on invariant pattern recognition by the visual system, combined with wavelet analysis using Krawtchouk functions as the mother wavelet. Our method precisely identifies the localization and frequency characteristics of the impulse components in EEG signals. Tested on task-related EEG data, it accurately detected blink components (0.5 to 1 Hz) and separated muscle artifacts (16 Hz). It also identified muscle response durations (298 ms) within the 1 to 31 Hz range in emotional reaction studies, offering insights into both individual and typical emotional responses. We further illustrated how the new method circumvents the uncertainty principle in low-frequency wavelet analysis. Unlike classical wavelet analysis, our method provides spectral characteristics of EEG impulses invariant to time shifts, improving the identification and classification of EEG components.

**Supplementary Information:**

The online version contains supplementary material available at 10.1007/s12021-024-09698-y.

## Introduction

When analyzing EEG signals, it is crucial to have information about their impulse components, such as blinks, muscle artifacts, bursts of brain activity, etc. At the same time, information about the time of impulse appearance, i.e., its localization and frequency characteristics, which allow its identification, is essential for further analysis. For example, in brain-computer interface (BCI) systems, it is necessary to identify local reactions for their further analysis or removal. These motor and other events are associated with focal changes in the brain activity and spatio-temporal patterns of EEG (Guger et al., 2000). Also, determination of the localization of the impulse component is necessary in studying the dynamics of processes, not only their average characteristics. Unfortunately, Heisenberg’s principle for Fourier analysis states that it is impossible to determine both the exact location of an impulse and its frequency features. Applied to Fourier analysis, it means that the greater the concentration of a function in space, the more “smeared” and uncertain its Fourier transform should be Bonami et al. (2003). The Fourier transform is a traditional method for identifying a signal’s spectral properties. Applying the Fourier transform is based on the assumption that the signal is stationary and ergodic (Trethewey, 2003). However, this allows estimating the signal characteristics only in average but not the dynamics. One approach for analyzing non-stationary signals is to use a windowed Fourier transform. While using this method a non-stationary signal is pre-divided into segments (windows) where the signal is considered as stationary. However, the Heisenberg uncertainty principle for Fourier analysis (Bonami et al., 2003) makes it impossible to determine time and frequency simultaneously. We can only state that the coordinate resolution of a windowed transform is determined by the width of the window function and is inversely proportional to the frequency resolution (Mallat, 2009). For the Fourier and the windowed Fourier transforms these restrictions are fundamental (Bonami et al., 2003). Fast Fourier Transform (FFT) for signal processing can be particularly problematic when the signal consists of randomly occurring transients superimposed on a more continuous signal (Trethewey, 2003). Unfortunately, for example, most of the signals resulting in the brain are non-stationary and non-ergodic. Applying the classical spectral analysis and the Fourier transformation in the window to study the dynamics of the signals negatively affects the results. If the task is to investigate the reaction in the brain, it is necessary to extract information about the time and the frequency of some events in the signal. In this context wavelet analysis turned up as a solution to the problem of pinpointing local events. Morle and Grossman (1982-1984) (Mallat, 2009) proposed to use wavelet transforms of nonstationary signals. Here the non-stationary signal is decomposed into certain basic functions (other than harmonic), which are obtained from a prototype function by compression and shift. The prototype function is called the mother (basic) wavelet (Mallat, 2009). Usually one of following functions is used as mother wavelet: Mexican Hat, Daubechies Wavelet, Haar Wavelet, Morlet Wavelet and others (Mallat, 2009; Walnut, 2002). Among the most effective wavelets we find the Hermitian wavelets - a family of continuous wavelets, which are defined as the derivative of a Gaussian distribution (Mehra et al., 2018). These wavelets are very suitable for the analysis of signals containing some peaks, for example for the detection of singularities generated by localized defects in a mechanical system (Li et al., 2011; Zhang et al., 2018; Deng et al., 2017; Li & Zhang, 2020). But Hermitian functions are functions of a continuous argument which leads to difficulties in their application when implementing appropriate algorithms. In this context we propose to use Krawtchouk functions (Vainerman & Filimonova, 1994; Vainerman, 1992) as mother wavelets. Krawtchouk functions are the discrete analogue of Hermitian functions by using a simple idea: since polynomials are completely determined by their values at a sufficiently large number of different points, it is possible to define orthogonality (and also orthonormality) relations on certain polynomials by using only a discrete finite set of points. Their application to automated spectral analysis of arbitrary signals is free from the drawbacks which appear while using polynomials as functions of a continuous argument. Thus, our proposal to use the discrete Krawchouk functions as wavelets in signal processing is an advantage over classical approaches, but does not yet solve all problems. Also, wavelets fit into the principle of uncertainty because a basic wavelet is defined on a short time interval which corresponds to high frequency. When the wavelet is stretched this time interval gets longer and the frequency decreases (Mallat, 2009; Vošvrda & Schurrer, 2015; Bonami et al., 2003). This is also true for Hermite wavelets (Bonami et al., 2003; Ricaud & Torrésani, 2014; Wigderson & Wigderson, 2021).

Thus, the Heisenberg uncertainty principle does not allow solving the problem of simultaneously determining the localization of the impulse component of the signal and its frequency characteristics either by classical Fourier analysis or wavelet analysis. Both of these methods have fundamental limitations when trying to give precise answers to the questions “What?” and “When?”. However, our visual system is perfectly capable of answering the questions “What?” and “Where?” which, for one-dimensional signals, is equivalent to the question “When?” (Graumann et al., 2022). Therefore, we propose the next approach. In the first stage, we propose building a mathematical model of pattern recognition invariance by a visual system. We plan to incorporate this model into wavelet analysis at the next stage. We hypothesize that integrating the model of extraction of shift-invariant signal features into wavelet analysis will allow us to solve the problem of simultaneously accurate computation of the time coordinate of a local impulse in EEG signal and its frequency features. This integration will enable us to bypass the limitations imposed by the Heisenberg uncertainty principle.

## The Model of Shift Invariant Image Recognition of the Visual System

The problem of extracting a complete system of invariant features of a signal arises in signal processing (including images) as well as in automatic pattern recognition or automatic classification and diagnostics. It is necessary to separate the information about the characteristics of the signal from the information about the transformations that this signal has undergone. These transformations (e.g. shift, image rotation, scale conversion, etc.) cannot be controlled, a visual system must identify the image independent from its location, and the transformations should not affect the performance of the system. Therefore, the images that pass into each other under some specific transformations must be classified as equivalent.

It should be noted that the human or mammalian visual system to some extent is capable to extract the invariant features of a signal. It took some effort to understand the functions necessary for visual form perception. Based on the knowledge about the receptive field (RF) reactions and about the visual system in Glezer (1995); Kulikowski and Bishop (1981); Lindeberg (2013); Shapley and Lennie (1985); Schnitzler (1976) a theory was developed according to which the visual system performs spatial-frequency image filtering. To adapt this effect to general signal processing we have to understand how this filtering works. Let us consider in more detail the visual path from the retina to the cerebral cortex of primates. Visual information enters the retina and is transmitted to the brain through about a million nerve fibers that are united in the optic nerve (Jeffries et al., 2014; Hubel & Weisel, 1977). Most of the optic nerve fibers reach without interruption two cell nuclei that are located deep in the brain. These nuclei are called lateral geniculate nuclei (LGN). In turn, neurons of the LGN send their axons directly to the primary visual cortex (Jeffries et al., 2014; Ghodrati et al., 2017).

The RF of a retinal ganglion cell refers to the synaptic network of photoreceptors, bipolar, horizontal, and amacrine cells which come together to this one ganglion cell (Hubel & Weisel, 1977). A concentric RF has the central zone where the receptors are stimulated to give response and a peripheral inhibitory ring (off-on), or conversely, an inhibitory central zone and a peripheral ring that gives response (on-off). Concentric fields are used to describe the image by points (Hubel & Weisel, 1977). Mathematically, the spatiotemporal RF is defined by an impulse response function (weight function) describing the firing-rate response to a tiny spot which is on for a very short time (Mobarhan et al., 2018). Some methods of modelling this weight functions were developed with difference of arouse and inhibitory Gaussians (Mobarhan et al., 2018; Eiber et al., 2018; Einevoll & Heggelund, 2000; Lindeberg, 2021; Jacobsen et al., 2016; Bertalmío, 2019), with Gabor elements (Paik & Ringach, 2011; Lee et al., 2016; Cope et al., 2009) and others (Li et al., year; Bertalmío, 2019).

Retinal ganglion cells project sensory information to the lateral geniculate neurons of the thalamus. LGN neurons replicate the center-surround structure of their presynaptic partners (Hubel & Weisel, 1962). Yet, this does not mean that the thalamic fields are direct copies of those in the retina. A single LGN neuron might receive inputs from multiple ganglion cells, hence the spatial information sent from retina is remixed (Alonso et al., 2006). One LGN neuron is overlapped with the ON (OFF) sub-region of the RF of retinal cells according to feedforward or feedback excitation (Usrey et al., 1999; Lian et al., 2019). Hence RFs of the LGN describe spatial patterns of light and dark regions around an average illuminance level in the visual field (Hubel & Weisel, 1977; Lian et al., 2019; Liu et al., 2021). The neural networks of the visual cortex do not present a contour picture of the incoming image which exists on the retina (Glezer, 1995). For effective processing of incoming signals it is therefore necessary to reduce the information redundancy, which, based on known studies, can be assumed to be realized in the LGN (Zabbah et al., 2014). Let us look into more details of this process.

### Step 1

The first transformation of information in the visual system is already threefold: informations are transformed from photoreceptors through horizontal cells to bipolar cells (Barnes et al., 2020). The optical system of the eye projects the image on the retinal layer of photoreceptors, while a horizontal cell summarizes the arousal of a large number of photoreceptors. Then the average of the summarized signals is subtracted (using the reverse suppressed signal) from the signals coming from the photoreceptors to the bipolar cells (Yeonan-Kim & Bertalmío, 2016). This is because the horizontal cells relate the photoreceptors and bipolar cells with sufficient long connections which are parallel to the layers of the retina (Glezer, 1995; Hubel & Weisel, 1977). The human eye can operate in a wide range of illumination changes: about 11 orders, whereas separate neurons in the retina and also cells on a more higher layer may change the activity within only 2 orders. This small change is one of the components of the adaptation process to the level of illumination (Zapp et al., 2022). After the adaptation a new zero level is created (Barnes et al., 2020). The distribution of the illumination can now be described in terms of areas which are brighter or darker relative to the average level (Glezer, 1995; Yeonan-Kim & Bertalmío, 2016; Barnes et al., 2020). Hence, in the first step we characterize the field of vision by *M* input signal points $y_\text {input}(i)$, $i=1,\ldots , M$. We model the distribution function *y*(*i*) of illumination transmitted to the receptive fields of the retinal ganglion cells (the first transformation of the signal) by

$$\begin{aligned} y(i)=y_\text {input}(i) -\overline{y_\text {input}},\ i =\overline{1,M}, \end{aligned}$$

where $\overline{y_\text {input}}=\sum y_\text {input}(i)/M$ is the average of the illumination for the visual field. Contacts of bipolars with ganglion cells are executed by amacrine cells that play the role of interneurons, and under transition from bipolars to ganglion cells the arousal transforms the analog impulse to a digital one, which justify the use of discrete functions to simulate the processes in the visual system. Applying this transformation to the incoming signal, we provide the invariance property under illumination variations (Lindeberg, 2013).

### Step 2

The concentric RF responds with a certain number of impulses per unit time (Hubel & Weisel, 1977) (with discrete functions). Consequently, an adequate simulation should use functions of discrete arguments. The LGN is increasingly regarded as a “smart-gating” operator for processing visual information in which the relatively elaborate features are computed early in the visual pathway (Tang et al., 2016). It was shown that at high contrasts neurons of LGN tuned to higher frequencies, which enhances the use of high frequency in the analysis of images (Glezer, 1995). As contrast increases, the RFs overlap and the response function becomes alternating with respect to the average illuminance level (Glezer, 1995). For efficient coding in digital image processing linear filters should be orthogonal (Cho & Choi, 2014). Therefore, in Cho and Choi (2014)it was proposed to describe the RF of a retinal ganglion cells in terms of an orthogonal wavelet basis. Since the structure of the LGN inherits the structure of the retina RF, we can assume the efficiency of using orthogonal basis functions for modeling the LGN RF.

Using experimental data regarding the visual system (Glezer, 1995; Hubel & Weisel, 1977) it was suggested to describe the weight functions of the RF of the LGN with Krawtchouk functions (Vainerman & Filimonova, 1994; Vainerman, 1992). Polynomials which are orthogonal on the set of points $\{1,\ldots N\}$ with respect to the binomial distribution were introduced by Mykhailo Krawtchouk, hence the denomination Krawtchouk functions. They are also called normalized Krawtchouk polynomials (Vainerman, 1992; Al-Utaibi et al., 2021; Jahid et al., 2017; Karmouni et al., 2017) in analogy with Hermite functions referring the fact that Krawtchouk polynomials are the discrete analogues of Hermite polynomials (Krawtchouk, 1929; Area et al., 2014), or weighted Krawtchouk polynomials ( Venkataramana and Raj, 2011; Idan et al., 2020; Yap et al., 2003), weighted and normalized Krawtchouk polynomials (Zhang et al., 2010; Abdulhussain et al., 2018; Asli & Flusser, 2014) or Krawtchouk functions (den Brinker, 2021).

To give a precise description of these functions we recall the definition of the binomial distribution $\rho (i)\in \mathbb {R}$:

$$ \rho (i) = \frac{N!}{i!(N-i)!} p^{i}(1-p)^{N-i}, \ i=1,2,\ldots ,N, $$

where $N\in \mathbb {N}$ and $p\in \mathbb {R}$ with $0< p <1$ are given beforehand. We introduce following notation for the Krawtchouk polynomials: $K_{n}^{(p)}(i,N)$, here *n* is the order of the polynomial and *p* plays the role of a coefficient of asymetry. To compute Krawtchouk polynomials we can use the following recurrent relation (Vainerman, 1992):

$$\begin{aligned} K_{1}^{(p)}(i,N) =&1;\ \ \ K_{2}^{(p)}(i,N) = i-pN.\\ K_{n+1}^{(p)}(i,N) =&(i-n-p(N-2n))\frac{K_{n}^{(p)}(i,N)}{(n+1)}\\&- p(1-p)(N-n+1)\frac{K_{n-1}^{(p)}(i,N)}{(n+1)}, \end{aligned}$$

with $$ n,i=1,\ldots ,N$$.

The orthogonality relation for the Krawtchouk polynomials reads

$$\begin{aligned} \sum _{i=1}^{N} K_{m}^{(p)}(i,N)K_{n}^{(p)}(i,N)\rho (i) = \delta _{mn}N!\frac{p^{n}(1-p)^{n}}{n!(N-n)!}. \end{aligned}$$

For applications the property of symmetry is important:

1 $$\begin{aligned} K_{n}^{(p)}(i,N) = (-1)^{n}K_{n}^{(1-p)}(N-i,N). \end{aligned}$$

**Fig. 1:**
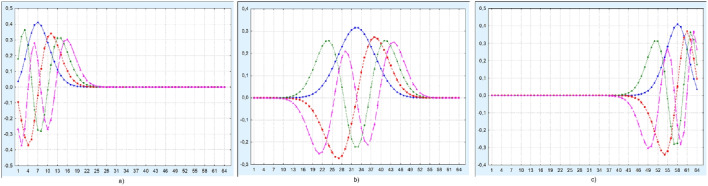
The first four Krawtchouk functions for values a) $p = 0.1$, b) $p = 0.5$, c) $p = 0.9$; $i = \overline{1, 64}$

By analogy with the Hermite functions, the corresponding Krawtchouk functions are determined as

2 $$\begin{aligned} F_{n}^{(p)}(i)=K_{n}^{(p)}(i)\sqrt{\rho (i)}/\sqrt{N![p(1 -p)]^{n}/n!(N-n)!}, \end{aligned}$$

where $N\in \mathbb {N}$ and $p\in \mathbb {R}$, $0< p <1$, are set in advance (Vainerman, 1992) (Fig. 1). Krawtchouk polynomials are orthogonal, but not normalized, however, for Krawtchouk functions we have

3 $$\begin{aligned} \sum _{i =1}^{N}F_{n}^{(p)}(i ,N)F_{m}^{(p)}(i ,N)=\delta _{nm}. \end{aligned}$$

We also obtain a recurrence relation for the orthonormalized Krawtchouk functions $F_n$:

$$\begin{aligned} F_{1}^{(p)} =&\!\sqrt{N !p^{i}(1 -p)^{N -i}/i !(N -i)i !},\\ F_{2}^{(p)} =&\!(i -qN)\sqrt{p^{i -1}(1 -p)^{N -i -1}(N -1)!/i !(N -i)!}, \\ F_{n+1}^{(p)} =&\!(i -n -p(N -2n))\frac{F_{n}^{(p)}}{\sqrt{(n+1)(N -n)p(1 -p)}}\\&- \sqrt{{(N -n+1)n \over (N -n)(n+1)}}F_{n -1}^{(p)}(i ,N), \end{aligned}$$

where $i = \overline{1,N}$, $n =\overline{3,N}$, $0< p <1$. In Al-Utaibi et al. (2021); Jahid et al. (2017); Karmouni et al. (2017); Idan et al. (2020); Yap et al. (2003); Zhang et al. (2010); Abdulhussain et al. (2018); Asli and Flusser (2014); Venkataramana and Raj (2011) recurrence algorithms for fast computation of the Krawtchouk functions are presented.

The symmetry relation Eq. 1 for the polynomials turns into an analogous formula for the Krawtchouk functions

4 $$\begin{aligned} F_{n}^{(p)}(i, N) = (-1)^ n F_{n}^{(1-p)}(N-i, N). \end{aligned}$$

In Fig. 1 the first four Krawtchouk functions $F_{n}^{(p)}$, $n=1,2,3,4$ are shown for different coefficients of asymmetry $p=0.1, 0.5, 0.9$.

**Fig. 2:**
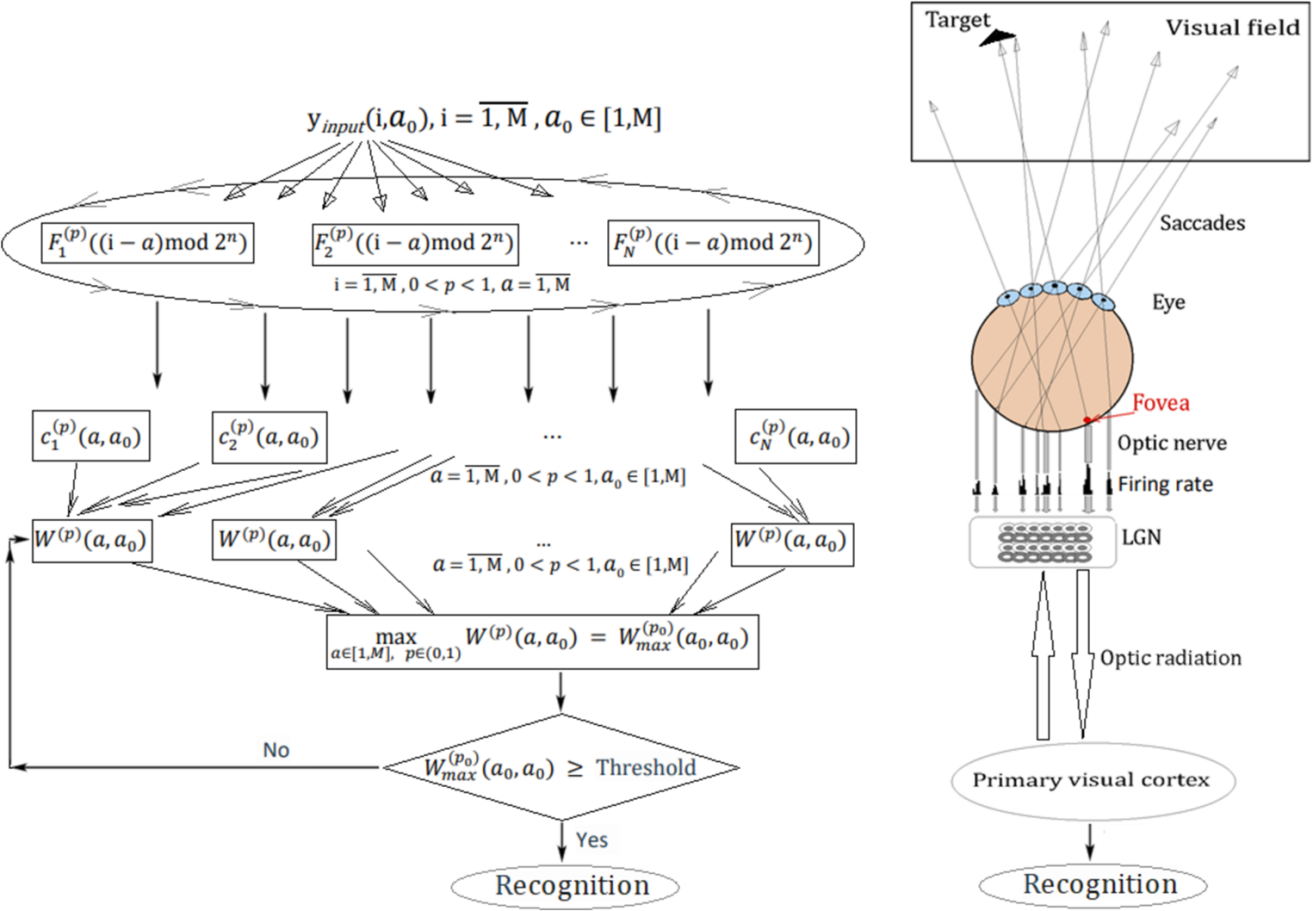
The inclusion of saccades in the model of the visual system

### Step 3

Mathematically, the model of the RF is defined by impulse response weight functions. Assuming linearity the response of the RF to any input signal *y*(*i*) can be found by convolving it with these weight functions (Mobarhan et al., 2018; Lindeberg, 2021; Liu et al., 2021; Cho & Choi, 2014). As mentioned in the previous step we model the weight functions with Krawtchouk functions. The main specific feature of our model is the introduction of the shift operator which we use to parameterize the basis functions. The idea is the following: the system receives a signal *y*(*i*) with an impulse component of unkown location $a_{0}$ as a hidden variable which will be determined later with our algorithm. We calculate the generalized spectral coefficients of the signal $y(i,a_{0})$ related to a set of basis functions $\{F_{n}^{(p)}(i-a, N),n= \overline{1,N}\}$ for all possible values $a =\overline{1,N}$ of the shift operator. Applying a standard procedure from the theory of image recognition we glue the ends of the visual field. The novelty of our approach lies in the resulting set of linear transformations by using the functions $F_{n}^{(p)}((i-a)^ {*}\text{mod}\,N, N), a =\overline{1,N}, 0< p < 1$. In this case, we will get the maximum response exactly when the value of the shift *a* coincides with the hidden value $a_{0}$ or, more precisely with the temporary location of $y(i,a_{0})$ (Fig. 2).

In addition, the Krawtchouk functions contain the parameter *p*, which determines their degree of asymmetry and allows us to find the basis with the best match to the shape of the signal $y(i,a_{0})$. In the visual system, the shift of the concentric RF (accordingly, basis functions) throughout the visual field can be related to eye saccades. Eye saccades are fast eye movements that provide an oriented shift of the gaze toward the location of an object. By positioning the image of an object on the retinal region of highest acuity - the fovea, the eye saccades increase the number of neurons activated by the presence of the object; a recruitment which likely contributes to improve its identification and localization (Goffart, 2017; Sarrabezolles et al., 2017) (Fig. 2). The eye saccades depend on the relative properties of the visual targets (e.g. size and energy), their landing position adjusts to the larger or to the brighter target in the visual field (Heeman et al., 2014). Thus, among all possible responses of saccadic eye movements, one can choose the position of RF oriented to the larger or to the brighter target with the maximum energy. We include saccadic eye movements in our visual system model because it makes it possible to separate information about the location of an object from its shape, in other words it ensures the invariance of object recognition regardless of its location (Fig. 2). The site of saccadic suppression is likely to be the LGN (Ghodrati et al., 2017), which agrees completely with our model. We assume that saccades are not so much suppressed in the LGN as they are used to determine the position of an object, which exhausts their role in the processing of visual information.

Hence we end up with generalized spectral coefficients $c_{n}^{(p)}(a,a_{0})$ of the following form

$$\begin{aligned} c_{n}^{(p)}(a ,a_{0}) = y(i,a_{0})*F_{n}^{(p)}((i-a)^ {*}\text{mod}\,N, N), \end{aligned}$$

here $i,a,n =\overline{1,N},0<p <1$. Here, with $*$ we refer to the discrete convolution.

### Step 4

Krawtchouk functions form orthonormal sequences. The idea is to determine the signal $y(i,a_{0})$ as a finite linear combination of Krawtchouk functions with suitable values *a* and *p*. To this end we define the energy functional

5 $$\begin{aligned} W^{(p)}(a,a_{0})=\sum _{n =1}^{N}\vert c_{n}^{(p)}(a,a_{0})\vert ^{2}. \end{aligned}$$

According to Bessel’s inequality we have

6 $$\begin{aligned} \sum _{n =1}^{N}\vert c_{n}^{(p)}(a,a_{0})\vert ^{2} \le \Vert y \Vert ^{2}. \end{aligned}$$

In Eq. 6 equality holds if and only if $y(i,a_{0})$ belongs to a linear envelope of $F_{n}^{(p_{0})}((i-a_{0})^{*}\text{mod}\,N,N)$. Since $W^{(p)}(a,a_{0})$ is nonnegative Eq. 5 and bounded Eq. 6 its supremum exists and will be attained when the variable parameters will coincide with the hidden ones.

### Step 5

We determine a subspace $\mathcal {K}$ of dimension *K*, $K < N$, which is generated by the functions with the biggest spectral coefficients. In this step we can essentially limit the number of functions, since we only want to find the location of the signal. Here we simulate the process of saccadic eye movement to find the position of the image of the visual system in the field of view. At first, the visual system approximates the object and in a second step the object will be focused and considered in more detail. In this step we find the low spatial frequencies (the ’fuzzier’ parts) of the image to understand where the image is. In the next step we will find the higher spatial frequencies, the fine details of an image. For the low frequencies we determine some bound $r,\ r\in \mathbb {R}$ for the spectral coefficients $\{c_{n}^{(p)}(a,a_{0}) \ge r\}_{n\in \mathcal {K}}$ and build the energy functional

7 $$\begin{aligned} W^{(p)}(a,a_{0})=\sum _{n\in \mathcal {K}}\vert c_{n}^{(p)}(a,a_{0})\vert ^{2}. \end{aligned}$$

We find the supremum of the energy functional

8 $$\begin{aligned} \text {sup}_{p\in (0 ,1),a\in [1,N]}W^{(p)}(a ,a_{0})=W^{(p_{0})}(a_{0},a_{0}). \end{aligned}$$

The hidden value of the shift $a_{0}$ is the value where the energy functional reaches its supremum. This location corresponds to the location of those RF which have maximum response. It gives the information about the location of the signal. The coefficient of asymmetry $p_{0}$ adjusts the shape of the Krawtchouk functions in the algorithm to the ratio between the front and back of the signal, and thus gives the information about the form of the signal. These two information about the location and form of the signal have been separated.

### Step 6

In the interactive mode with feedback, in each step we find the optimal set of generalized spectral coefficients $\mathcal {K}$ with the dimension $K < N$, which provide the restoration error $\varepsilon$ we set in advance. In this step, we simulate the feedback of the visual cortex to the LGN. The function of massive feedback projections from the striate cortex to the LGN, far superior to direct retinal projections, remains an unsolved mystery (Ghodrati et al., 2017). An explanation has been proposed that corticothalamic feedback improves feature recognition (Sillito et al., 1994; Zabbah et al., 2014). In Martinez-Canada et al. (2018) it was shown that in the presence of feedback the maximum RF of LGN response occurs to smaller stimuli, i.e. it is accompanied by spatial focusing. This explanation of the role of the visual cortex feedback to the LGN is fully consistent with our model, since at this step of the algorithm it allows us to include higher frequency Krawtchouk functions in the signal processing and identify more finer signal structures.

We define the restored signal

9 $$\begin{aligned} y^{\prime }(i):=\sum\limits _{n=1}^K{\,}c_{n}^{(p_{0})}(a_{0},a_{0})F_{n}^{(p_{0})}((i-a_{0}) ^{*}\text{mod}\,N),\ \ i =\overline{1,N}. \end{aligned}$$

The restauration error is then $\varepsilon = \Vert y^{\prime }-y\Vert$, where $\Vert \cdot \Vert$ is the Euclidean norm in $\mathbb {R}^{K}$. Thus, this set of generalized spectral coefficients contains the essential information of the signal form and represents the essential features of the signal. Also, these spectral coefficients are invariant to the hidden shift of the signal because we obtain in addition the shift $a_{0}$ and the coefficient of asymmetry $p_{0}$ and accordingly, the information of the form of the signal has been separated from its location.

In Usrey and Alitto (2015) it is shown that visual processing in the LGN includes spatial and temporal influences on visual signals that serve to adjust response gain, transform the temporal structure of retinal activity patterns, and increase the signal-to-noise ratio of the retinal signal while preserving its basic content. At the same time in Graumann et al. (2022) it was found that location representations of the objects emerge along the ventral visual stream towards the lateral occipital complex. Their calculation involves recurrent processing in the high-level visual cortex. Our model is consistent with these results and allows us to assume an important role of the LGN in the recursive process of finding both informative features of objects and their location by the visual system. As usual models of technical systems are robust, but this model of a biological system is sensitive to small details of the image. As eyes can see small image features so also our proposed algorithm can extract the corresponding features. This property can be useful, for example, in automated ECG diagnostic systems, since the small details such as “little plateaus”, “little jags” etc. are significant for diagnosis, but most existing algorithms smooth them out. The problem of model robustness is solved on the stage of decision making: the investigator decides to take these details into account or to reject them as noise.

A convolutional neural network (CNN) was recently developed as a model of the visual system, where each neuron processes the input signal exclusively within its receptive field (Rakhmatulin et al., 2024). Consequently, our proposed method can be naturally understood within the framework of CNNs. These networks have been successfully applied to EEG analysis for recognizing various human states, including depression, neurocognitive dysfunctions, emotional states, and motor imagery for brain-computer interfaces. Additionally, CNNs have proven effective in decoding task-related information directly from raw EEG data without relying on handcrafted features (Rajwal & Aggarwal, 2023; Schirrmeister et al., 2017; Rakhmatulin et al., 2024). A typical CNN architecture consists of three layers: the Convolutional Layer, the Pooling Layer and the Fully-Connected Layer (Rajwal & Aggarwal, 2023). In the Convolutional Layer, the input signal is convolved with weight functions that model the responses (filters) of receptive fields, enabling the determination of the significant signal features for subsequent identification and classification. This feature extraction process is a critical step in EEG signal analysis, as it transforms raw data into numerical features suitable for further processing (Rakhmatulin et al., 2024). The quality of these features significantly impacts the signal identification and the classification efficiency in subsequent CNN layers. Consequently, this study focuses on identifying informative signal features that are shift-invariant and independ of the signal’s starting point. These informative signal features can be applied across various classification methods, though their analysis is beyond the scope of this article. As highlighted in Rajwal and Aggarwal (2023), certain challenges arise when using CNNs for EEG analysis. At first, CNNs require large amount of training data. Given that EEG recordings often have varying starting points and are inherently non-stationary signals, the potential size of the training dataset becomes virtually infinite. Our proposed method extracts invariant features, which can help reduce the size of the required training set. Another challenge in using CNNs for EEG analysis is overfitting, where the model becomes too closely tailored to the training data. compromising its ability to generalize to unseen data (Rajwal & Aggarwal, 2023). In our approach, the training sequence is utilized to determine the threshold for the maximum energy functional, which serves as the pooling mode. This threshold determines the accuracy of signal recovery, effectively defining the required level of signal detail. According to the maximum energy functional value, only features with amplitudes exceeding a certain threshold are considered significant. While higher-order frequencies typically exhibit lower amplitudes than low frequencies, they are crucial for capturing the more minor signal details. Consequently, increasing the number of Krawtchouk functions in the CNN Convolution Layer enhances the model’s ability to generalize to unseen data. Importantly, our approach does not rely on predefined signal features from the training set, but adapts the set of filters in the Convolution Layer to the signal shape. Importantly, our approach does not rely on predefined signal features from the training set to adapt the model to a new input signal. Instead, the Convolution Layer’s filters are dynamically adjusted to match the shape of the input signal. This key distinction from existing methods helps mitigate overfitting, as the training process is designed to be universal for signals of a specific type, independent of the exact values of the training features. Another challenge in using CNNs is the complexity of interpretation (Rajwal & Aggarwal, 2023). Typically, it is unclear what specific features a CNN utilizes for classification. In our proposed method, however, the set of informative features is entirely transparent, consisting of signal decomposition coefficients derived from Krawtchouk basis functions. These functions are optimally adapted to the localization of the signal’s impulse components and its asymmetry.

**Fig. 3:**
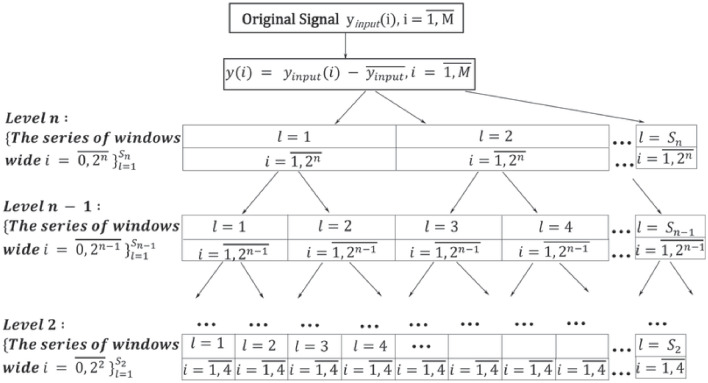
Signal wavelet transformation scheme

In our opinion, a more significant issue with applying CNNs to EEG signals is their non-stationary nature (Rajwal & Aggarwal, 2023). In the CNN algorithm, the Convolution Layer processes the input signal within a fixed window, convolving it with filter weight functions across all frequencies. If the processing window is determined by the wavelength of the lowest frequency, higher frequencies are also confined to this window. As a result, high-frequency components, which may undergo multiple oscillations within the window, can exhibit time-varying dynamics that the CNN algorithm cannot effectively capture. While CNNs excel at visual image analysis (Rakhmatulin et al., 2024), they are not inherently designed to analyze signal dynamics. To address this limitation, we incorporated wavelet analysis into our approach. At the next stage, we developed a method that combines a Convolutional Layer for extracting invariant signal features with signal analysis performed within wavelet analysis windows of varying scales.

## Algorithm for Determining the Temporal Localization of the Impulse Components of the Signal and their Frequency Characteristics

**Fig. 4:**
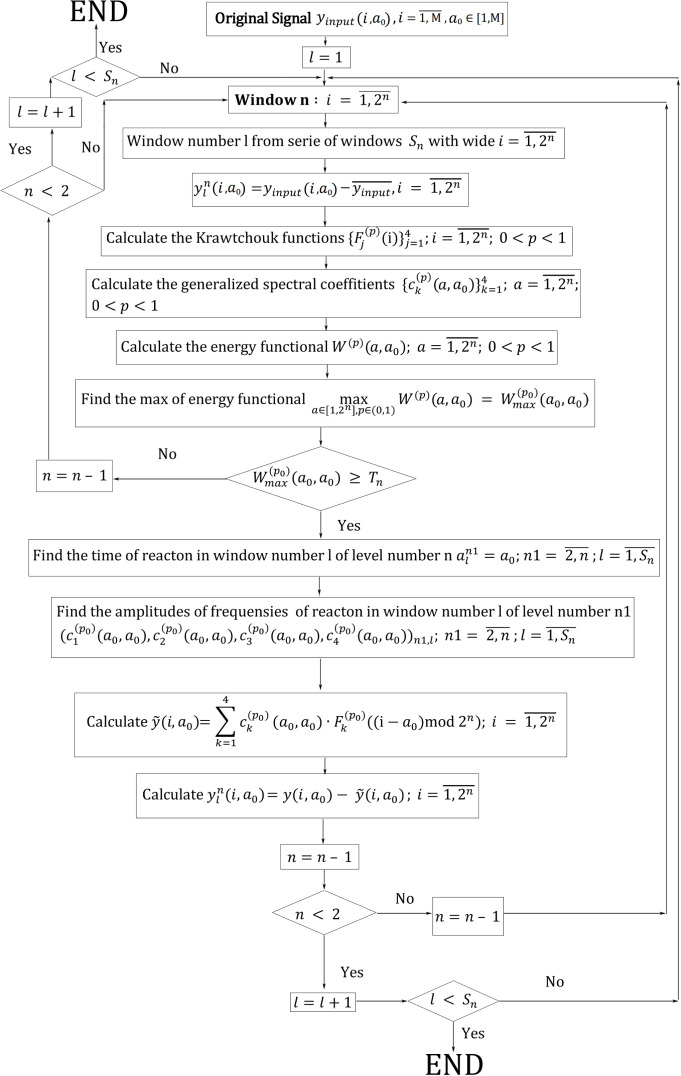
The algorithm of signal processing

The ideology of the wavelet transform is to consequently decompose a signal with some impulse functions in different scales (Fig. 3). In each cell of this scheme we will use an algorithm based on the model presented above (Fig. 4). We propose a wavelet transform with Krawtchouk functions as the mother-wavelet, based on ideas from image recognition. The input of the system receives a signal $y(i,a_{0})$ with an impulse component localized in the short duration of the continuous signal which corresponds to some reaction of the process under study. We will discuss what is meant by the reaction in Step 7 of the next algorithm. The task is to find the reaction time (the time of the impulse component) and determine its frequency characteristics.

### Step 1

We define the scale of the wavelet transform with window widths of *N* points, $N =2^{n},n =10,9,\cdots ,2.$ We begin from window widths of 1024 points because we use this algorithm to analize the EEG which was recorded with a discrete frequency of 500 Hz and with filters of high and low frequencies respectively with 0,5 Hz and 200 Hz.

### Step 2

We create a new zero level of the input signal in the biggest window: $y(i,a_{0})=y_{input}(i,a_{0}) - \overline{y_{input}(i,a_{0})}, i =\overline{1,1024}$ where $\overline{y_{input}(i,a_{0})}=\sum y_{input}(i,a_{0})/1024$ is the average of illumination for the biggest window.

### Step 3

We calculate the first four Krawtchouk functions for the windows $N =2^{n},n =10,9,\dots , 2$ and $p=0.1, 0.2,\dots , 0.9$, since we use 9 asymmetry coefficients of the Krawtchouk functions for a better approximation of non-symmetrical impulse components. Since the functions are symmetric Eq. 4, we only need to calculate the Krawtchouk functions for $p=0.1, 0.2, 0.3, 0.4$ and for $p=0.6, 0.7, 0.8, 0.9$. At $p=0.5$ the Krawtchouk functions are symmetric w.r.t. the middle of the interval, therefore, they are calculated only for $i=0, 1,\cdots , N/2$. This reduces the accumulation of the error in the recurrent calculation of the following function values. Fast algorithms for the computation of the Krawtchouk functions can be found in Al-Utaibi et al. (2021); Jahid et al. (2017); Venkataramana and Raj (2011); Yap et al. (2003); Zhang et al. (2010); Abdulhussain et al. (2018); Asli and Flusser (2014). In addition, we can calculate the function values in advance and save them in the corresponding file. In each window we use the first four Krawtchouk functions, since numerical experiments which analyze various signals have shown that this is the optimal number of functions. The use of a smaller number of functions does not allow a sufficient analysis of the shape of complex impulse components, and a larger number of functions captures the frequencies of the next level. Also, the size of the last smallest window is four points. Thus, the four Krawtchouk functions in the last window constitute a complete set of basis functions, which makes it possible to carry out an exhaustive analysis of the signal and avoid losses.

### Step 4

We calculate the generalized spectral coefficients relative to the set of shifts of the basis of Krawtchouk functions: $c_{k}^{(p)}(a,a_{0}) = y(i,a_{0})*F_{k}^{(p)}((i-a)^{*}\text{mod}\,N)$, where $i,a=\overline{1,N}; k = 1,2,3,4; p=0.1, 0.2,\dots , 0.9$. To calculate the generalized spectral coefficients, we use the convolution theorem

$$\begin{aligned}c_{k}^{(p)}(a,a_{0})= &  y(i,a_{0}) *F_{k}^{(p)}(i -a)\\= &  \mathcal {F}^{ -1}[\mathcal {F}(y(i,a_{0}))\cdot \mathcal {F}(F_{k}^{(p)}(i - a))], \end{aligned}$$

where $\mathcal {F}$ is the Fourier transformation, $i =\overline{1,N},k =\overline{1,4}, p=0.1, 0.2,\dots , 0.9$. We keep in mind that the ends of the window of view have been glued.

### Step 5

We calculate the energy functional

$$\begin{aligned} W^{(p)}(a ,a_{0})=\sum _{k=1,2,3,4}\vert c_{k}^{(p)}(a,a_{0})\vert ^{2}. \end{aligned}$$

### Step 6

We find the maximum of the energy functional

$$ max_{p=0.1,\dots , 0.9; a=\overline{1,N}}W^{(p)}(a,a_{0})=W_{max}^{(p_{0})}(a_{0},a_{0}).$$

At this step, we find the temporal localization of the impulse component of the signal and the asymmetry coefficients of the Krawtchouk basis functions, which maximally correspond to the asymmetry of the impulse. Convolutional neural networks are very effectively used to analyze and classify EEG, for example, in Lo Giudice et al. (2023). In contrast to convolutional neural networks, we use a convolution of the input signal with a set of bases, which is parameterized by the shift and asymmetry parameters rather than a single basis. At this step, we find the unique basis in which the impulse has a response with the highest energy.

### Step 7

Now we determine whether the extremum found in the previous step has to be interpreted as a “reaction” or as background fluctuations. If $$W_{max}^{(p_{0})}(a_{0},a_{0}) \ge T_{n}$$, where $$ T_{n}$$ is the threshold value of the energy functional for the window of level *n*, we define this part of the signal as reaction and go to the next step. The threshold values $$T_{n}$$ are found in advance using a training sequence. This training algorithm of classifiers finds the $$min_{d=\overline{1,D}}W_{max,d}^{(p_{0})}(a_{0},a_{0})=T_{n}$$, where $$d=\overline{1,D}$$ is a set of signals with an impulse component, which the experts define as a “reaction”, $$W_{max,d}^{(p_{0})}(a_{0},a_{0})$$ are the energy functionals of the “reaction”. In the case $$W_{max}^{(p_{0})}(a_{0},a_{0}) < T_{n}$$ we proceed to signal processing inside the windows of the next level.

### Step 8

We determine the time of reaction (impulse) in window number *l* of level number *n*: $a_{l}^{n1} = a_{0}$, $n1 = \overline{n,2}$; $l = \overline{1,S_{n}}$ and find the amplitudes and frequencies of reaction (impulse) in this window: ${c_{k}^{(p_{0})}(a_{0},a_{0})}_{k=1}^{4}$, $n1 = \overline{n,2}$.

### Step 9

We restore the impulse component of the signal

10 $$\begin{aligned} \tilde{y}(i)=\sum_{k=1,2,3,4}c_{k}^{(p_{0})}(a_{0},a_{0})F_{k}^{(p_{0})}((i-a_{0})^{*}\text{mod}\,N), i =\overline{1,N}. \end{aligned}$$

### Step 10

We subtract the reconstructed signal from the input signal: $y_{l}^{n} = y(i,a_{0}) - \tilde{y}(i); i = \overline{1,2^{n}}$. Thus, we consider oscillations at low frequencies as drift of the isoline for the oscillations of high frequencies. In addition, this eliminates the repeated finding of the frequency characteristics of the same impulse component in the next windows of the wavelet analysis. This approach is also one of the distinguishing features of our algorithm compared to other wavelet transform algorithms.

### Step 11

We go to the next window $l = l+1$ at this level *n* of wavelet analysis. If the rest of the signal is less than the window width, we pad the signal with zeros: if $M < (S_{n}-1) \cdot 2^{n}$, then $y(i) = 0, i = \overline{M,S_{n} \cdot 2^{n}}$. In case we have reached the end of the sequence of windows of a given level $l > S_{n}$, we go to the next level of windows $n = n-1$. In the case $n < 2$ the algorithm reaches the end.

When estimating the time complexity of an algorithm, only the component with the fastest growth rate is considered. In the case of our algorithm, the most computationally expensive operations are the recursive calculations involved in generating the Krawtchouk functions. To optimize performance, we precomputed these functions and their Fourier transforms, storing them in corresponding files for reuse. The next most significant contributors to time complexity are typically loop executions and procedure calls. For our algorithm, this includes the convolution computations, which we optimized by employing the Fast Fourier Transform (FFT) procedure to reduce computational overhead. We used the Cooley-Tukey FFT algorithm, which has a time complexity of $O(N \log _2 N)$, where $N = 2^n$ (with *n* representing the maximum power of 2 defining the largest scale in wavelet anaysis) is the length of the largest wavelet analysis window.

**Fig. 5:**
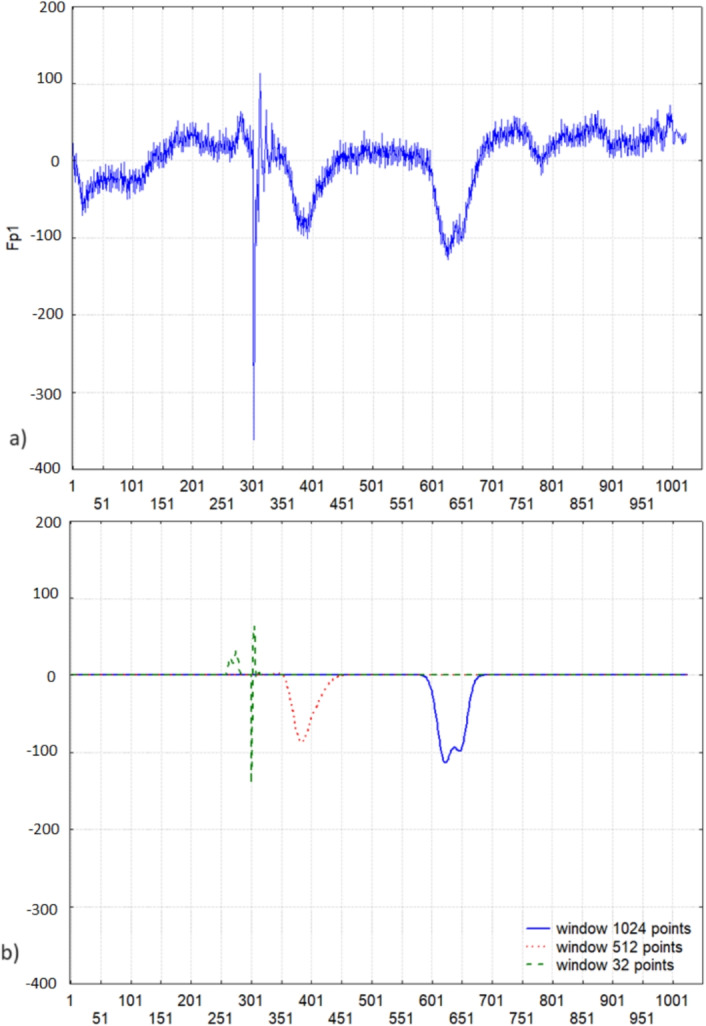
The fragment of an EEG recording in the lead Fp1 a) with the events of blinking and a muscle artifact and b) its restorations according to the formula Eq. 10

The next most computationally intensive component is the set of nested loops in our algorithm. Specifically, our algorithm employs five nested loops, resulting in a time complexity of $O(n1\cdot 4\cdot p1 \cdot N\cdot N)$. Here, *n*1 represents the number of scale levels, *p*1 is the number of asymmetry coefficient values *p* for the Krawtchouk functions, *N* is the length of the largest window, and the second *N* corresponds to the number of shifts applied to the Krawtchouk functions. For instance, EEG data was recorded at a sampling rate of 500 Hz, using a high-pass filter at 0.5 Hz and a low-pass filter at 200 Hz. For analysis, we employed windows with a maximum length of $N =1024 =2^{10}$, $(n=10)$, with $p1 = 9$ asymmetry coefficients $(p=0.1, 0.2,..., 0.9)$. The first four Krawtchouk functionswere utilized at each of $n1 = 9$ scale levels $(k = 10, 9,..., 2)$. When calculating the complexity, constant multipliers can be disregarded, simplifying the algorithm’s complexity to $O(N \log _2 N) + O(N^2)$. Based on wavelet analysis principles, long signals are processed sequentially in windows of maximum size. As a result, the algorithm demonstrates manageable complexity and is well-suited for real-time EEG analysis, even with larger datasets.

For comparison, we outline the time complexities of the CNN method and wavelet analysis. For a CNN architecture, the algorithm’s complexity can be approximated as $O(k\cdot N \cdot M \cdot nF^{L-1} \cdot nF^L)$, where *k* is the size of the kernel matrix, *N* and *M* are the number of rows and columns of the input matrix, $nF^{L-1}$ represents the number of filters in the preceding CNN layer and $nF^L$ is the number of filters in the current layer (Rusnac & Grigore, 2022). In contrast, the time complexity of the wavelet transformation for a signal of length *N* is *O*(*N*) (Amin et al., 2020).

## Experimental Results

### Example N1

When studying the processes of attention, it is important to be able to analyze the moment of blinking. As a rule, in this case, minima are found in the electrical activity records of the brain in the prefrontal leads. However, muscle artifacts may be present in these recordings. Currently, they are found and removed from further analysis manually. We applied the developed method to find the moments of blinking and will use it in future to recognize and remove the muscle artifacts automatically from further analysis. Figure 5 a) shows the moments of blinking on the scales of 1024 and 512 points. The EEG was recorded with the sampling rate of 500 Hz, so the blinking moments are defined at 346 points = 653 ms and 513 points = 1026 ms. Thus, blinking was identified in the range of 0.5 - 1 Hz, and the muscle artifact was identified in the range of 16 Hz (32 points).

The proposed method allowed not only the calculation of the exact time coordinates of different impulse components but also their frequency characteristics, which were used to reconstruct the corresponding impulses according to the formula Eq. 10 (Fig. 5 b)). This proves that our hypothesis about the method of integrating the model of extraction of shift-invariant signal features into wavelet analysis is confirmed. Thus, the calculated generalized spectral coefficients contain information about the impulse shape. They can be further used to identify and classify different impulses and decide whether to remove or include them in subsequent analyses. For example, blinks can be used to study attention processes or simply removed from the EEG signal.

We performed a statistical analysis comparing the positions of the maximum energy and signal extremes functions for 25 different EEG recordings with localized impulses. A distribution of the absolute values of the difference between the maximum energy and signal extremes was significant differing from the normal distribution (Shapiro-Wilk’s test $$ W = 0.432514$$; $$p < 0.001$$). In this case, a valid estimate of central tendency is the median Me, which here is Me $$=8$$ points, and the variance is represented by the interquartile range [Lower Quartile, Upper Quartile], which is Guger et al. (2000); Cope et al. (2009) points. The inexact coincidence of the maximum energy functional and the momentum extrema is explained by the fact that the energy functional is a smooth function and the momentum extrema may have local point-like outliers.

### Example N2

In a study of human emotional reactions, an EEG was recorded with sampling rate of 500 Hz during the presentation of pictures. The pictures were randomly selected from a database of standardized sets of pictures, the International Affective Picture System (IAPS). In addition to brain activity, the researchers were interested in muscle reactions in certain leads upon presentation of some pictures. The task was to find the time, duration and power of the muscle reaction of individual subjects during the presentation of certain pictures.

**Fig. 6:**
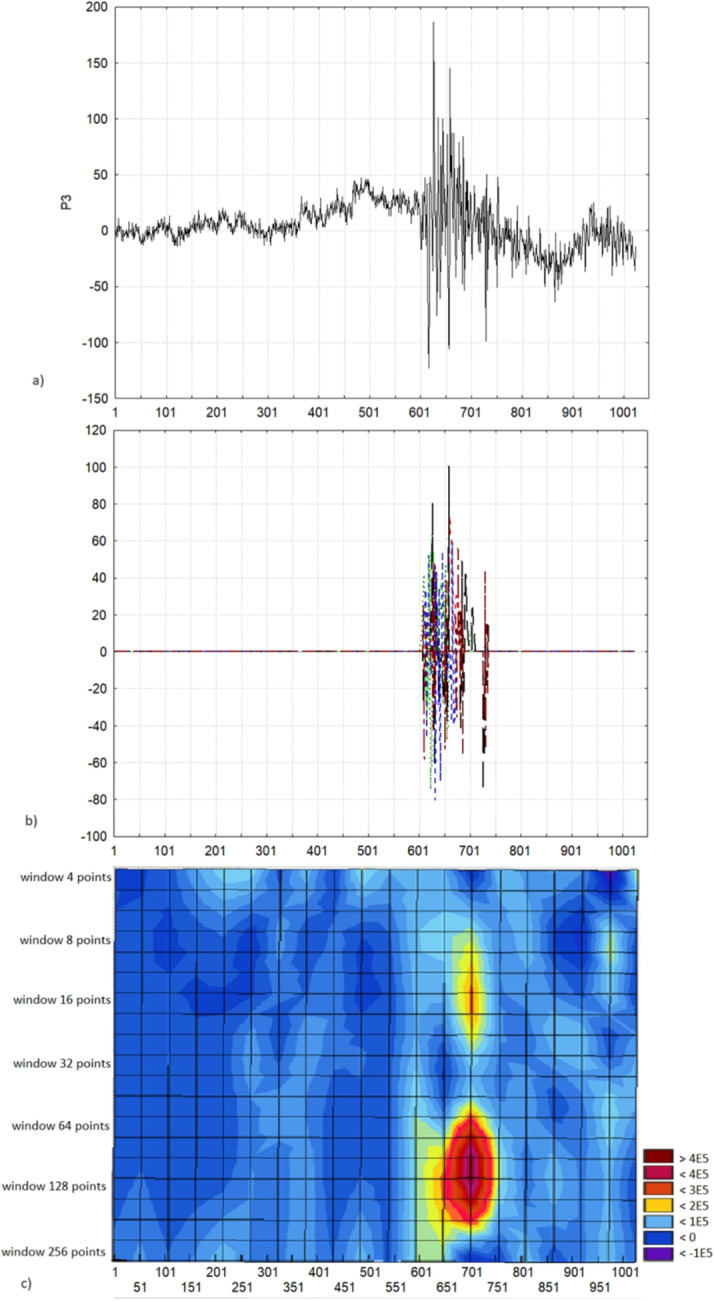
The fragment of an EEG recording in the lead P3 with a) the muscle reaction when viewing an emotional picture, b) the localization of the muscle reaction - the restored signal by the corresponding generalized spectral coefficients in different frequencies according to the formula Eq. 10 and c) its wavelet spectrum

Figure 6 shows the results of processing the EEG record in lead P3 when viewing a certain picture in one subject. The muscle response was found in the range of scales 512 - 16 points (1 - 31 Hz) between the points 587 - 736 (1174 - 1472 ms). Thus, the duration was 298 ms with a power of $\sum _{n=\overline{9,5}, l=\overline{1,S_{n}}} W^{(p_{l})}_{n}(a_{l},a_{l})= 1979384\ \text {mkV}^2$. This makes it possible to compare the presence/absence of a muscle reaction in a given subject to various pictures and the presence/absence of a typical muscle reaction in other subjects to similar pictures.

It is noteworthy that the proposed method made it possible to calculate not only the time parameters of the muscle reaction but also the frequencies, which further opens up the possibility of determining the features of both individual and typical emotional reactions.

### Example N3

Removal of the artifacts from EEG is a large topic that requires detailed discussion. We plan to demonstrate the capabilities of our method for removing artifacts of different types in EEG in the next article. Now we would like to highlight that certain artifacts can carry additional useful information. Usually, the researcher is not able to analyze artifacts, because existing methods reject them and the researcher analyzes only the clean EEG in the next step. In the following example, we want to demonstrate that decomposing the initial EEG into artifact and clean EEG can provide additional possibilities for analyzing human brain activity.

**Fig. 7:**
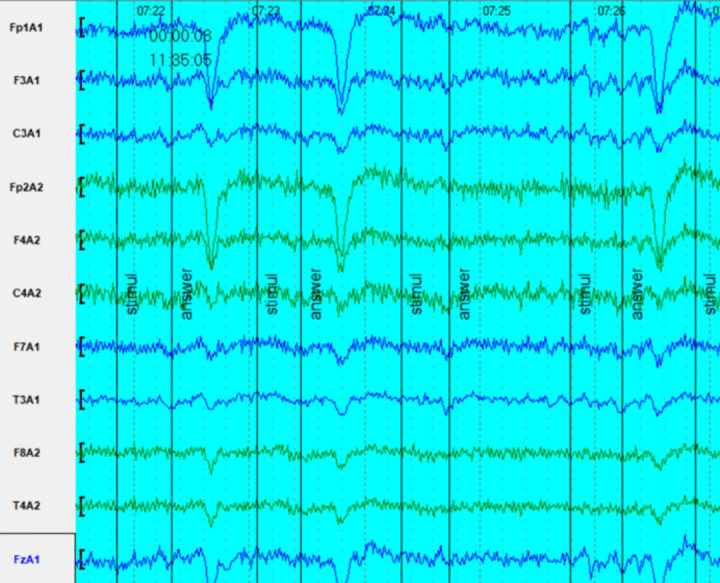
The fragment of an EEG recording during the Choice Reaction was examined

In this example, we consider a study in which the Choice Reaction was examined. In this case, the EEG was labeled “stimulus” during stimulus presentation, and the EEG was labeled “answer” when a button was pressed during the person’s response. As can be seen from Fig. 7 blinking is often associated with the moment of response. This may be a demonstration of a generalized motor response that may be of interest in BCI studies.

**Fig. 8:**
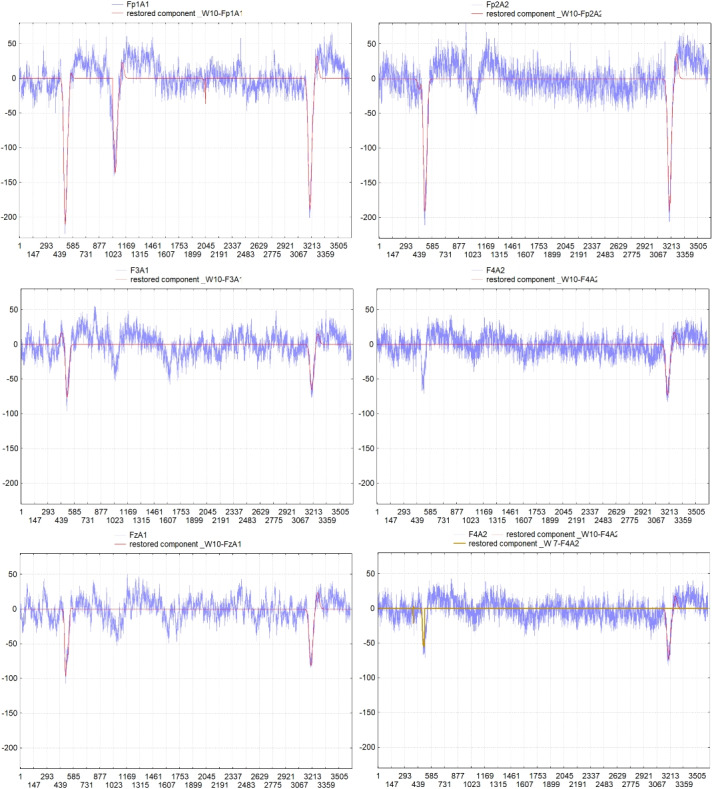
The fragment of an EEG recording during the Choice Reaction was examined

In Fig. 8 we have presented the decomposition components of EEG recordings in frontal leads in the largest window of wavelet analysis of 1024 points. The thresholds were chosen in such a way as to extract blinks in the largest window of wavelet analysis and find temporal parameters of their localization. Some blinks were lost, for example in lead F4, because their amplitude was not enough to cross the corresponding threshold. But we find this impulse on a different scale - in a 27 = 128-point window. This example shows the difficulties we have with finding a balance between the thresholds in different scales. But this example also demonstrates the potential of the method for studying synchronization processes in brain activity during test tasks.

**Fig. 9:**
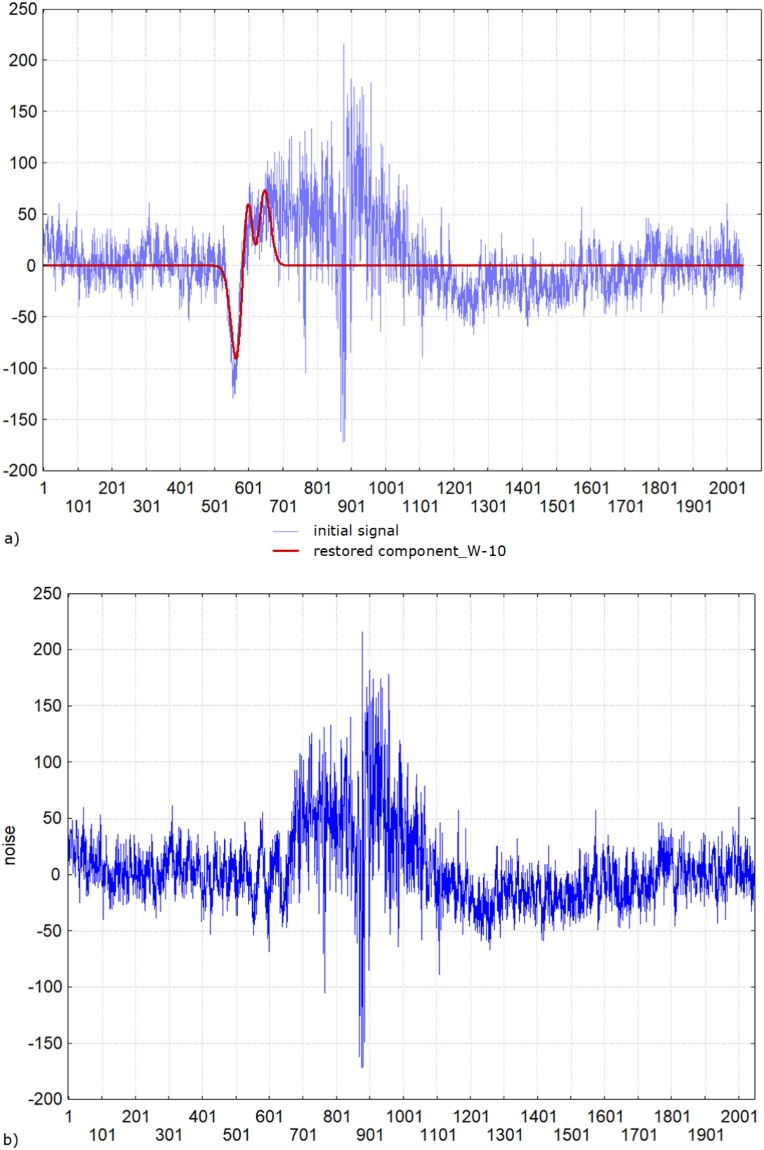
a) Initial EEG recorded in Fp1A1 and the first component of the wavelet analysis of it in the largest window of 1024 points W-10, which was calculated according to the formula (9); b) the noise

### Example N4

**Fig. 10:**
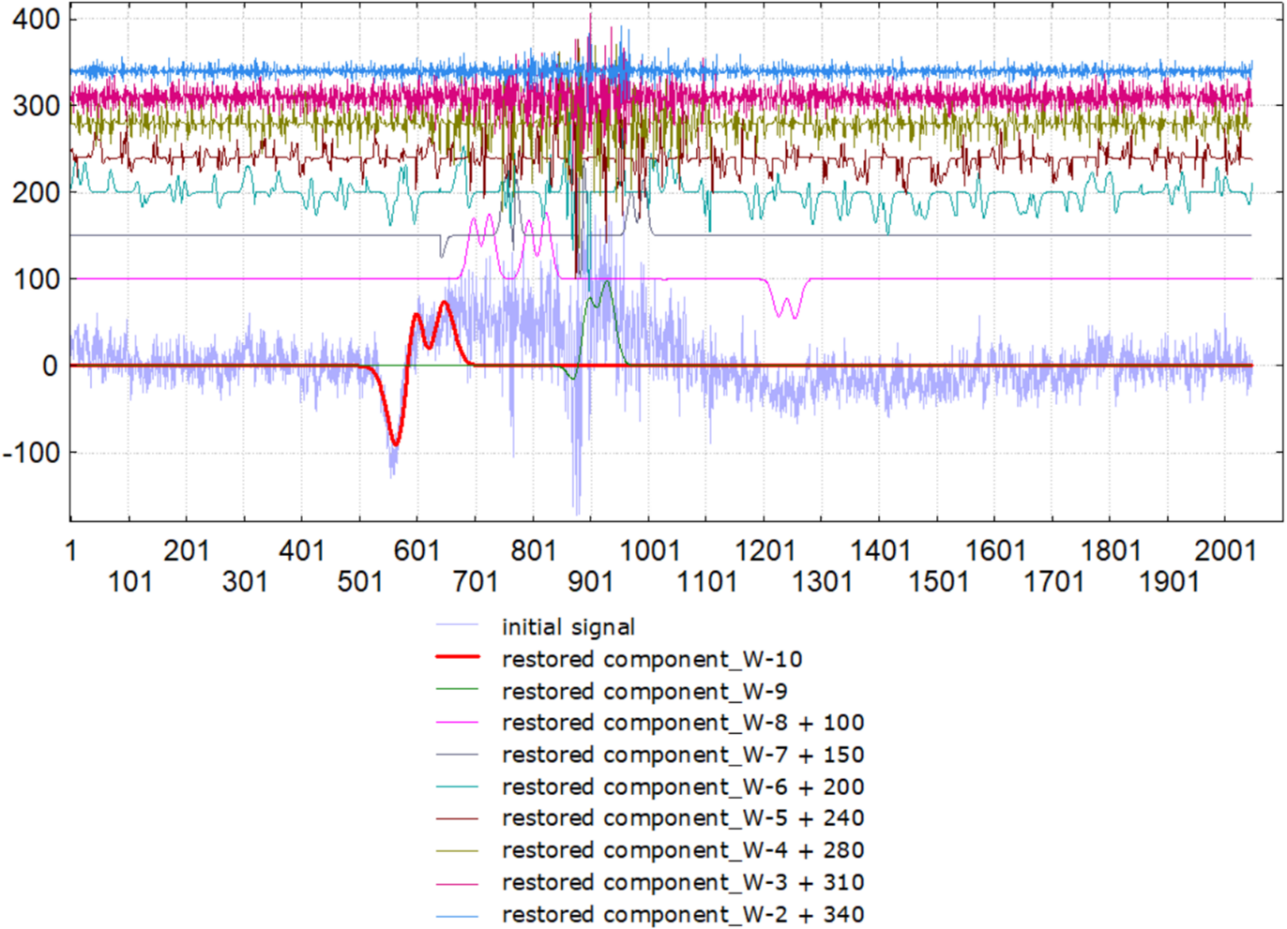
Initial EEG recorded in Fp1A1 and its decomposition in all wavelet windows from 1024 points to 4

In EEG analysis tasks, it is often necessary to separate the signal from the background noise. In the following example, we will demonstrate the capabilities of the method to extract the impulse component in Fp1A1. Using the impulse component, we will understand an impulse associated with blinking. The extraction of such a component may be of interest in the tasks of BCI systems. Figure 9 a) shows the original signal-EEG recording in the Fp1A1 lead with the overlaid component, which was extracted in a window of 1024 points according to formula (9). Figure 9 b) shows the noise graph. Note that the impulse component was extracted despite the presence of concomitant high-frequency and high-amplitude noise. Noise we define as sum of components in the windows from 4 to 512 points. In the Fig. 10 we present the decomposition of initial signal in all wavelet windows from 1024 points to 4. The graphics of the components, which were restored in the windows from $2^8=256$ to $2^2=4$ we presented with the shift in the y axis for better visualization. The energy of the extracted signal was calculated using the formula $E(\text {W-10}) = \sum _{i=1}^{1024} \sum _{j=1}^4 |c_j|^2(i) = 193050614,065$. The noise energy was calculated using the formula $E(\text {noise}) = \sum _{k=2}^9\sum _{i=1}^{1024}\sum _{j=1}^4 |c_j^k|^2(i) = 204026625,049$. So, the signal/noise ratio is 0.95. Let us define the distance between the initial signal and the sum of the impulse component and noise as the maximum of the absolute difference of ordinates, then it is equal to 0.00001. It proves that all information without any losses is contained in the components of the wavelet decomposition, and the researcher can decide for himself what information should be saved and analyzed in the future and what should be rejected.

### Example N5

We determined the physiological price paid by the organism during cognitive load based on the analysis of the electrocardiogram (ECG) recorded during the test tasks. The goal was to determine the degree of tension in the regulatory mechanisms of the human organism. To do this, we calculated the stress index (SI) according to Baevsky, which determines the ratio of activity of the sympathetic and parasympathetic divisions of the autonomic nervous system (Baevsky & Chernikova, 2017).

**Fig. 11:**
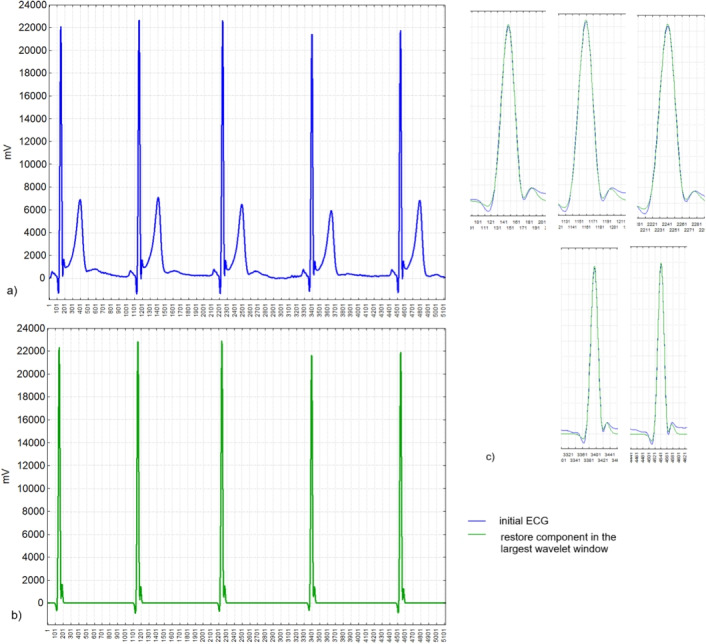
Initial ECG and the first component of the wavelet analysis of it in the largest window of 1024 points, which was calculated according to the formula (9)

The first step was to determine the heart rhythm based on the initial ECG. As shown in Fig. 11, the first component of the wavelet analysis of the initial ECG in the largest window of 1024 points, which was calculated according to the formula (9), allowed us to determine the location of the R wave of the QRS complex with an accuracy of 1 point. Since we recorded the ECG with a sampling rate of 1000 Hz, the accuracy of determining the R-R intervals was 0.001-0.002 s.

**Fig. 12:**
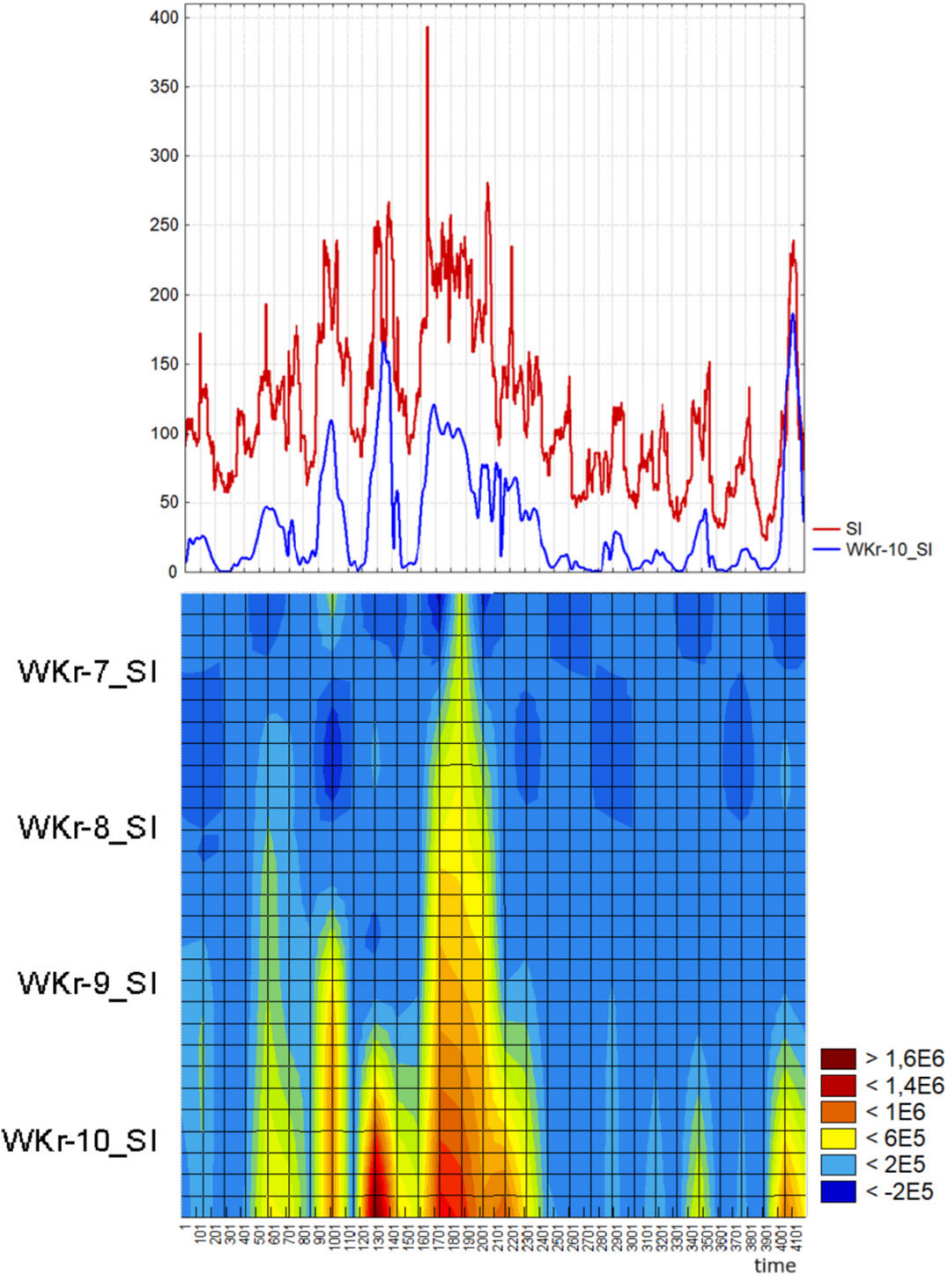
The stress index of the driver during the test on the simulator with different levels of the stress load, the first component of the 1024-point window wavelet spectrum WKr-10$\_$SI, which was calculated according to the formula (9) and the wavelet spectrum of SI

The dynamics of the SI for a car driver was studied. Test recordings of the ECG were carried out in collaboration with TROUT GmbH (2022) in participants while passing specific modes of the car driving simulator. On the basis of ECG analysis, the SI was calculated in dynamics with a shift of one second. The task was to determine the time of change in the driver’s state by the level of his SI. Since SI has many outlying points and numerous local maxima, it was difficult for us to determine the threshold that would indicate a permanently elevated level of SI. Therefore, we started looking for a parameter that would be smoother, correlated well with SI, and that we could analyze in run-time. We used the wavelet analysis for the SI and its wavelet spectrum is shown in Fig. 12 with windows SI$\_$1 with 1024 points, SI$\_$2 with 512 points, ..., SI$\_$5 with 64 points. The SI variables and amplitudes of the wavelet spectra had a distribution which is significantly different from Gaussian (according to Lilliefors test $p<0.01$), so we evaluated their interrelation by the Spearman correlation coefficient. The SI correlated with the first component of the 1024-point window wavelet spectrum, which we calculated according the formula (7), with $r_s = 0.84$. On this basis, we could find times of changes in the driver’s state during the test with different stress loads.

## Wavelet Analysis with Krawtchouk Functions as Mother Wavelet

We want to show the difference between the proposed method, which we will call wavelet analysis for localization, and the classical wavelet analysis, which uses Krawtchouk functions as the mother wavelet. Let us consider the wavelet analysis method where the mother wavelet is defined by Krawtchouk functions. According to Walnut (2002), the wavelet analysis algorithm consists of the following steps.

### Step 1

We define the scale of the wavelet transform with the windows of length of *N* points, $N =2^{n},n = 2,3,\dots ,10$. In the wavelet analysis method using invariance with respect to shift proposed above, which we will call wavelet analysis for localization, we used the first four Krawtchouk functions in each analysis window. Therefore, in this algorithm of wavelet analysis without invariance, which we will call wavelet analysis, we will also use the first four Krawtchouk functions in each window. For the first four Krawtchouk functions, we define the scaled and translated basis functions $F_{k}^{n}(i), k = \overline{1,4}; i = \overline{1,N}$ for the windows $N =2^{n},n = 2,3,\dots , 10$ and $p=0.5$. The parameter *n* defines the scale (or dilation), parameter *b* the location (shift, translation) of the wavelets which changes discretely proportional to the n-th degree of the wavelet scale $b = b_{0}*s$, where $b_{0} = 2^{n}, s = \overline{0,2^{10-n}}$, then $F_{k}^{n,b}(i) = F_{k}^{n}(i + b), i = \overline{1,1024}$.

### Step 2

We define a signal processing window with a maximum degree of two, determining the segment of the signal to be analyzed. In this instance, we select the initial segment of the signal, denoted by *y*(*i*), with a length of $N =2^{n} = 1024, n = 10$ points. Subsequently, we proceed to the next segment and continue this process until reaching the end of the signal.

### Step 3

We start the signal processing with the smallest windows with a width of $N =2^{n} = 4, n = 2$ points.

### Step 4

The wavelet transform is computed by taking the inner product of *y*(*i*) with translated and scaled versions of a wavelet, as described in Burrus et al. (1997). The wavelet coefficients are determined through the inner product of signal and wavelet basis functions

$$\begin{aligned} c_{k}^{n,b} = \sum _{i = \overline{1,N}} y(i)*F_{k}^{n,b}(i), k = \overline{1,4}. \end{aligned}$$

### Step 5

We calculate the energy functional

$$\begin{aligned} W^{n,b} = \sum _{k=1,2,3,4}\vert c_{k}^{n,b}\vert ^{2}. \end{aligned}$$

### Step 6

We proceed to the next window with a length of $N = 2^{n}, n = 2$ points. We repeat steps 4-5 until we reach the end of the maximum-length window, $N = 1024 = 2^{n}, n = 10$.

### Step 7

We repeat steps 4-5 for windows with a width of $N =2^{n} = 8, n = 3$ points until we reach the end of the maximum-length window $N = 1024 = 2^{n}, n = 10$. Subsequently, we repeat steps 4-5 for windows with widths of $N = 2^{n}, n = 4, \cdot , 10$ points.

### Step 8

We proceed to analyze the next segment of the signal using the window of maximum length, following steps 2-7. If the remaining portion of the signal is smaller than the window width, we pad the signal with zeros. The algorithm is finished when the last segment of the signal has been analyzed.

The majority of studies on EEG wavelet analysis concentrate on methods for classifying EEGs based on their wavelet spectrum (Gosala et al., 2023; Subasi, 2005; Amin et al., 2020; Raghu et al., 2019; Zahirovic et al., 2021). While a variety of functions are employed as the mother wavelet (Bajaj, 2020), in our opinion, insufficient emphasis has been placed on investigating how the properties of these functions influence the accuracy of EEG signal decomposition. It is important to note that the French hat wavelet, Mexican hat wavelet, Morlet wavelet (or Gabor wavelet), and first-order Gaussian wavelet do not constitute a basis in the strict mathematical sense. This means they cannot form a coordinate system suitable for decomposing the original signal. Haar and Walsh wavelets, while forming bases, are periodic signals, which makes their energy functional unsuitable for identifying impulse localization. Daubechies wavelets, on the other hand, provide a discrete basis with an impulse-like shape, but they are complex to construct, and their shift parametrization is not straightforward. The Hermite wavelet is the most suitable wavelet for continuous variables. However, as it is defined over a continuous domain, its implementation in computational algorithms requires discretization. This discretization introduces errors that compromise the orthogonality of Hermite functions as basis functions, resulting in some loss of accuracy in EEG signal decomposition. Furthermore, Hermite functions are inherently symmetric, meaning that representing an asymmetric signal requires combining two components with distinct frequencies, corresponding to the signal’s leading and trailing slopes. Krawtchouk functions are inherently constructed as an orthonormal system with a discrete argument, making them well-suited as a mother wavelet and free from the limitations associated with discretizing continuous functions. Additionally, the inclusion of a parameter to define the asymmetry coefficient enables more precise analysis of asymmetric signals.

## Comparison of the Methods

The main difference between the proposed wavelet analysis method for localization and classical wavelet analysis is that in classical wavelet analysis, a single basis function is used in the signal processing window to decompose the signal into the corresponding generalized spectral coefficients (determining the amplitudes of the corresponding frequencies). We use a set of basis functions, which is parameterized by a shift and an asymmetry coefficient. Our approach aims to find the single basis among these basis functions that must conform to the impulse component and to provide that the values of the shift and asymmetry parameters are most appropriate for the corresponding properties of the impulse.

In this section we compare the results of signal analysis using wavelet analysis and wavelet analysis for localization, as described in the main part of the article. In these examples, we will denote the energy functionals calculated in the windows with a length of $2^{n}, n = 10, \ldots , 2$ points according to wavelet analysis as $W-n, n = 10, \ldots , 2$ and as $WKr-n, n = 10, \ldots , 2$ according to wavelet analysis for localization.

**Fig. 13:**
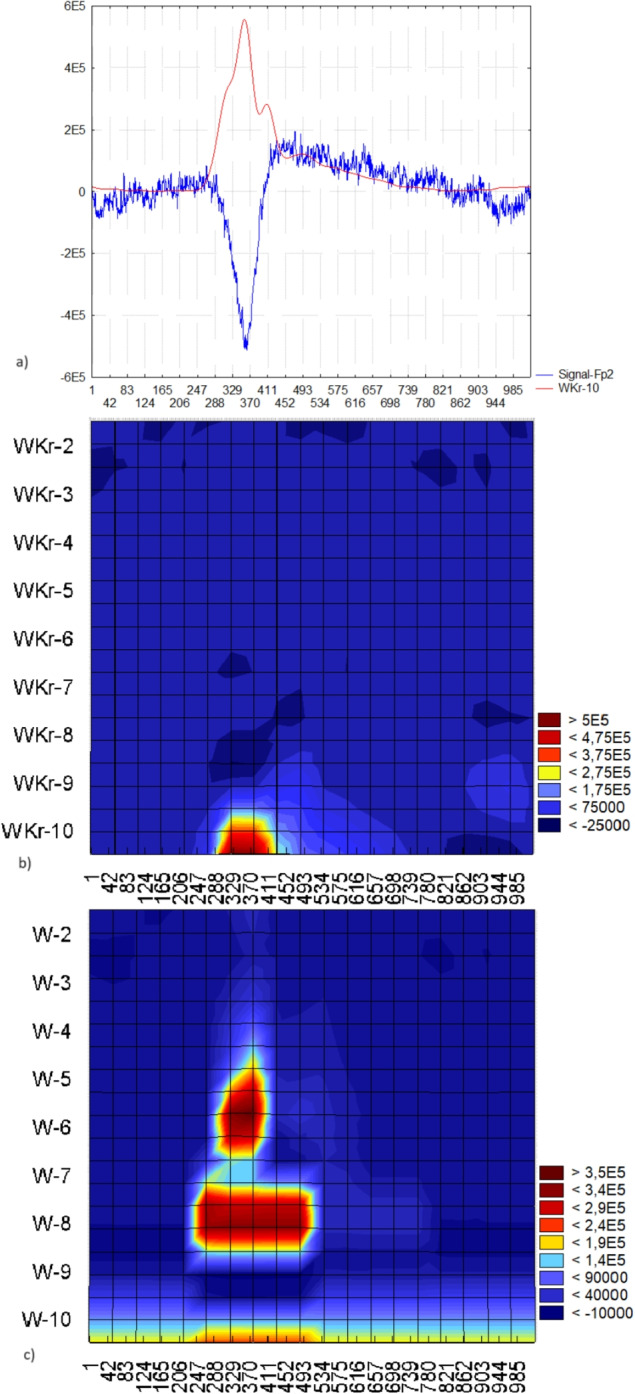
a) The segment of an EEG recording in lead Fp2 exhibiting ocular artifact and the superimposed energy functional $WKr-10$ within a window of 1024 points; b) wavelet spectrum derived from wavelet analysis for localization, and c) wavelet spectrum according to the wavelet analysis

Figure 13 displays a segment consisting of 1024 points from the EEG recorded in the Fp2 lead, featuring a blink artifact along with its wavelet analysis. In Fig. 13 a), the signal is presented with the superimposed energy functional $WKr-10$ in a window of length 1024 points. This example illustrates that the maximum of the energy functional aligns with the extremum of the signal. Figure 13 c) illustrates the uncertainty principle in wavelet analysis for low frequencies. Figure 13 b) shows that we have successfully circumvented the uncertainty principle, enabling simultaneous determination of signal localization and frequency analysis.

**Fig. 14:**
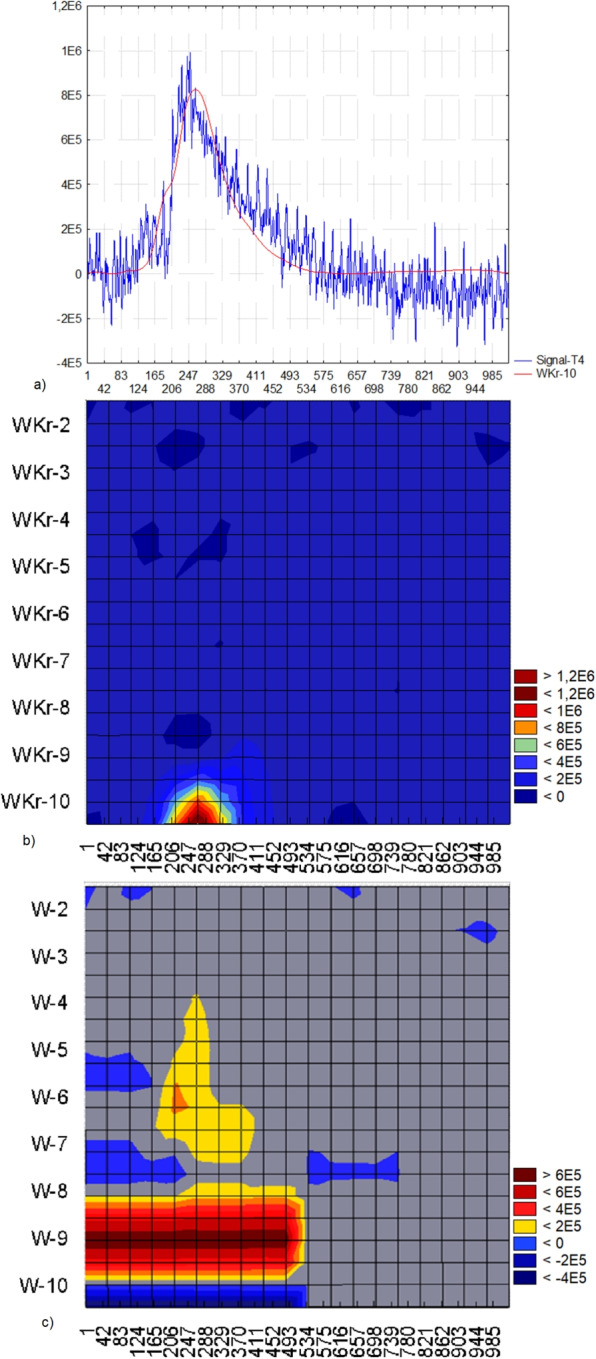
a) A segment of an EEG recording in lead T4 featuring the impulse component and the superimposed energy functional $WKr-10$ within a window of 1024 points; b) wavelet spectrum derived from wavelet analysis for localization, and c) wavelet spectrum obtained from the wavelet analysis

The maximum of the energy functional does not always precisely align with the extremum of the signal due to the smooth nature of the energy functional, which approximates the impulse. Additionally, the impulse may include a high-amplitude and high-frequency component. Figure 14 illustrates an example of an impulse where there is a 15-point difference between the extremum and maximum of the energy functional $WKr-10$.

**Fig. 15:**
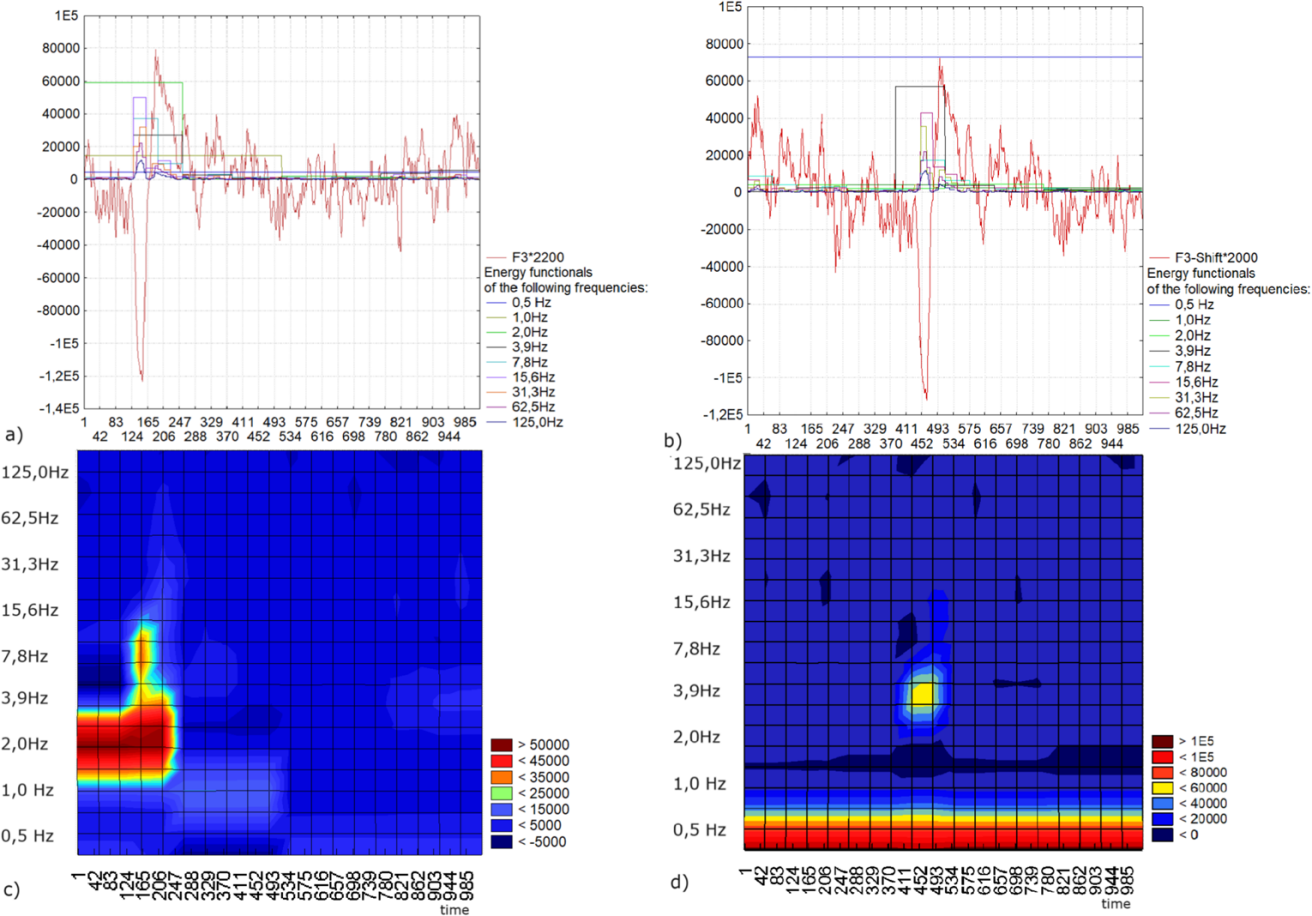
a) A segment of an EEG recording in lead F3 featuring the impulse component; b)a segment of an EEG recording in lead F3 displaying the shifted impulse component and the superimposed energy functionals for the windows $W-10, W-9, \ldots , W-2$ within the windows of $1024, 512, \ldots ,4$ points and in accordance of the frequencies 0,5 - 125 Hz; c) wavelet spectrum obtained from wavelet analysis for the signal a); d) wavelet spectrum according to the wavelet analysis for the signal b)

A limitation of spectral analysis, shared by wavelet analysis, is its susceptibility to variations based on the signal’s starting point. In this example, we demonstrate the invariance of the wavelet spectrum, generated using the proposed wavelet analysis method for localization, with respect to shifts in the impulse component of the signal. In classical wavelet analysis, the wavelet spectrogram of a signal is built by the scalar product of the signal with the daughter wavelets within the corresponding scale windows, determining the amplitude of the frequency components. In this case, the amplitude value is constant during the corresponding window Fig. 15 a) and b). Thus, for low frequencies (larger windows), according to the Heisenberg uncertainty principle, it is impossible to determine the localization of the impulse. Figures 13 - 15 c) and d) show the spectral powers of the signals, represented as the sums of the squares of the amplitudes resulting from the signal decomposition using the first four Krawtchouk functions within the scale windows W (k = 10, 9, ..., 2) with a length of 2n points (n = 10, 9, ..., 2). For this example, the wavelet spectrum was recalculated in Hz using EEG data sampled at 500 Hz. It is notable that the wavelet spectra for signals with a shifted impulse component exhibit significant differences, as illustrated in Fig. 15 c) and d).

**Fig. 16:**
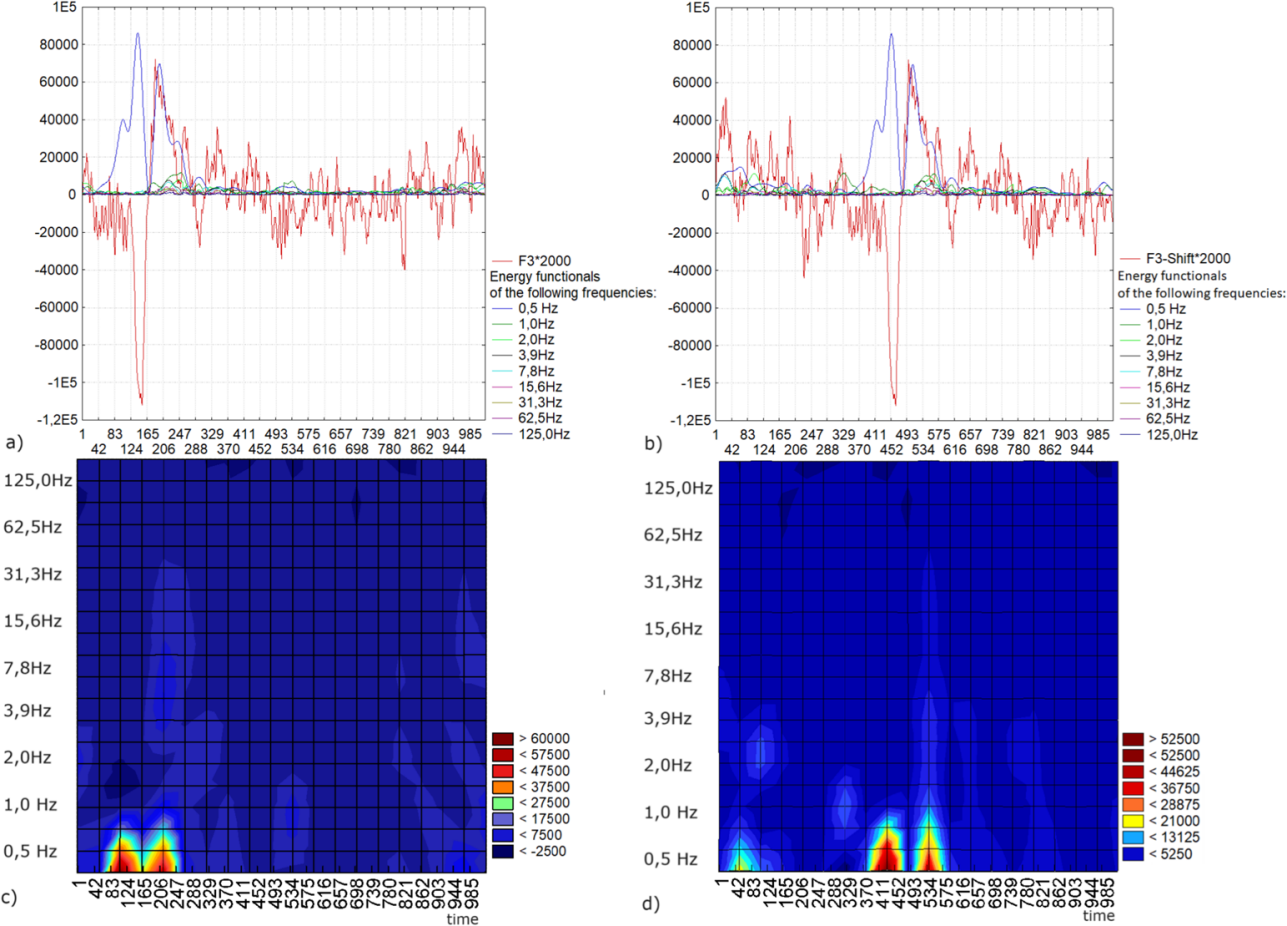
a) A segment of an EEG recording in lead F3 featuring the impulse component; b) a segment of an EEG recording in lead F3 displaying the shifted impulse component and the superimposed energy functionals for the windows $WKr-10, WKr-9, \ldots , WKr-2$ within the windows of $1024, 512, \ldots ,4$ points and in accordance of the frequencies 0,5 - 125 Hz; c) wavelet spectrum derived from wavelet analysis for localization for the signal a); d) wavelet spectrum derived from wavelet analysis for localization for the signal b)

In the context of wavelet analysis for the localization of the impulse component, we build wavelet spectrograms based on the energy functional. This functionals are defined as the sum of the squares of the convolution of the signal with the first four Krawtchouk functions, each shifted by the parameter $a_{0} (a_{0} = 0,1, \ldots , 2n,\ n = 10, 9, \ldots , 2)$ Fig. 16). As a result, the maximum of the energy functional coincides with the localization of the extremum signal peak. Figures 13, 14, 16 show the spectral powers of the signals in terms of the energy functionals within the scale windows WKr (k = 10, 9, ..., 2) with a length of 2n points (n = 10, 9, ..., 2). Figure 16 demonstrates that, as a result of wavelet analysis for localization, we obtain spectral features of the impulse component of the EEG signal that are invariant to the shift of this impulse.

The examples of numerical experiments fully confirm our hypothesis that the wavelet analysis method for localization allows us to simultaneously calculate both the time coordinates of an impulse and its frequency features.

## Conclusion

Our findings highlight the successful development of a method that simultaneously determines both the localization coordinates and frequency characteristics of a signal’s impulse component. This method integrates a model of invariant pattern recognition, inspired by the visual system, into our wavelet algorithm. For the first time, saccades are explicitly incorporated as a fundamental element of the visual system, providing the critical property of recognition invariance. By integrating the recognition invariance model with wavelet analysis, we developed an effective approach for analyzing non-stationary EEG signals with impulse components. This method was successfully applied to EEG experiments, demonstrating its capability for accurately determining both the precise localization and frequency characteristics of these impulse components. Furthermore, the generalized spectral features extracted from the impulse components can be utilized in machine learning algorithms, enabling efficient recognition and subsequent classification of EEG signals.

As we have demonstrated in the examples N3, N4, N5, our method is most successful in tasks where it is necessary to detect the impulse component of the signal or to detect the repetition of reactions that do not have a rigidly defined rhythm (example N3). The method allows us to decompose signals into components at different scales (N4), and to restore the initial signal as the sum of these components with any accuracy specified in advance. At the same time, the method underperforms for analyzing signals with insufficiently expressed changes in amplitude.

We plan to focus future research on developing a method for automatically removing artifacts from EEG signals, including single signals. In addition, based on this method, we plan to create a method for studying the dynamics of human brain connectivity using EEG recordings. We believe that this will enable researchers and medical experts to visualize the dynamics of existing neural networks and obtain information for analyzing multiple brain states and diagnosing neural network lesions. This method can be especially helpful in diagnosing invisible brain injuries on MRI and fMRI.

The most challenging aspect of our proposed method is determining the system of thresholds across different scales in wavelet analysis. Currently, these thresholds are established through extensive numerical experiments on signals of a specific type. In the future, we aim to develop specialized methods for training the algorithms using a designated training sequence.

We believe our contribution lays the groundwork for analyzing EEG signal dynamics, enabling the identification and classification of their impulse components. The capabilities of our method allow researchers to investigate non-stationary and transient processes in brain activity, as well as detect artifacts, anomalies, etc.

## Information Sharing Statement

The authors confirm that the data supporting the findings of this study are available within the article and its supplementary materials.

## Supplementary Information

Below is the link to the electronic supplementary material.

Supplementary file 1 (zip 772 KB)

## Data Availability

All data supporting the findings of this study are included in the supplementary material.
